# Personalized Breast Cancer Screening: A Risk Prediction Model Based on Women Attending BreastScreen Norway

**DOI:** 10.3390/cancers15184517

**Published:** 2023-09-12

**Authors:** Javier Louro, Marta Román, Nataliia Moshina, Camilla F. Olstad, Marthe Larsen, Silje Sagstad, Xavier Castells, Solveig Hofvind

**Affiliations:** 1 Department of Epidemiology and Evaluation, Hospital del Mar Medical Research Institute, 08003 Barcelona, Spain; jlouro@imim.es (J.L.); mroman@psmar.cat (M.R.); xcastells@psmar.cat (X.C.); 2 Network for Research on Chronicity, Primary Care, and Health Promotion (RICAPPS), 48902 Barakaldo, Spain; 3 Section for Breast Cancer Screening, Cancer Registry of Norway, 0304 Oslo, Norway; namo@kreftregisteret.no (N.M.); cfag@kreftregisteret.no (C.F.O.); maln@kreftregisteret.no (M.L.); sisa@kreftregisteret.no (S.S.); 4 Department of Health and Care Sciences, Faculty of Health Sciences, UiT, The Arctic University of Norway, 9037 Tromsø, Norway

**Keywords:** female, early detection of cancer, breast neoplasms, area under curve, retrospective studies

## Abstract

**Simple Summary:**

The study aimed to develop and validate a prediction model that can be used to classify women for tailored breast cancer screening based on their individual risk. The model included data on age, mammographic density, family history of breast cancer, body mass index, age at menarche, alcohol consumption, exercise, pregnancy, hormone replacement therapy, and benign breast disease for 57,411 women screened in BreastScreen Norway 2007–2019. The 4-year predicted risk of breast cancer ranged between 0.2% and 7.3%, with 95% of the population having a risk of 0.6–2.3%. The differences in the predicted risk favor personalized screening for breast cancer.

**Abstract:**

Background: We aimed to develop and validate a model predicting breast cancer risk for women targeted by breast cancer screening. Method: This retrospective cohort study included 57,411 women screened at least once in BreastScreen Norway during the period from 2007 to 2019. The prediction model included information about age, mammographic density, family history of breast cancer, body mass index, age at menarche, alcohol consumption, exercise, pregnancy, hormone replacement therapy, and benign breast disease. We calculated a 4-year absolute breast cancer risk estimates for women and in risk groups by quartiles. The Bootstrap resampling method was used for internal validation of the model (E/O ratio). The area under the curve (AUC) was estimated with a 95% confidence interval (CI). Results: The 4-year predicted risk of breast cancer ranged from 0.22–7.33%, while 95% of the population had a risk of 0.55–2.31%. The thresholds for the quartiles of the risk groups, with 25% of the population in each group, were 0.82%, 1.10%, and 1.47%. Overall, the model slightly overestimated the risk with an E/O ratio of 1.10 (95% CI: 1.09–1.11) and the AUC was 62.6% (95% CI: 60.5–65.0%). Conclusions: This 4-year risk prediction model showed differences in the risk of breast cancer, supporting personalized screening for breast cancer in women aged 50–69 years.

## 1. Introduction

In recent decades, the risks of mammographic screening, such as false positive screening results and overdiagnosis, have been debated [1,2,3,4]. Consequently, the current breast cancer screening strategy has been questioned at the same time as more personalized approaches have been promoted [5,6,7,8]. This might include moving from a one-size-fits-all strategy, a universal approach with the same screening interval and screening technique for all women within a defined age range [9], to recommendations determining interval, techniques, and age range according to the women’s individual risk of breast cancer. However, the implementation of personalized strategies poses the challenge of effective stratification based on the women’s risk of the disease.

Several risk prediction models [10,11,12] have been developed since the Breast Cancer Risk Assessment tool was presented as the basis for the first breast cancer prevention trials in the 1980s in the U.S. [13,14,15,16]. Despite a large number of models and substantial improvements in methodology, there is a need for risk assessment models since most of them were not specifically created for women targeted by screening and are not calibrated by the different incidences in the populations [17]. For this reason, at the 2019 European Conference on Risk-Stratified Prevention and Early Detection of Breast Cancer, experts on breast cancer screening stated the need to create and validate risk models based on data from large cohorts [18].

To meet this need, we aimed to develop and validate a breast cancer risk prediction model targeting women participating in population-based screening, using data from BreastScreen Norway.

## 2. Materials and Methods

This study has a legal basis in accordance with Articles 6 [19] (e) and 9 [20] (j) of the GDPR. The data was disclosed with legal bases in the Cancer Registry Regulations section 3-1 and the Health Register Act section 19 a to 19 h [21,22]. Data from women with negative screening results who have made a reservation against permanent storage of their personal data in the Norwegian Cancer Registry are not included in the dataset, cf. Cancer Registry Regulations section 1-9 [21] and the Personal Health Data Filing System Act, section 11 [23].

BreastScreen Norway started in 1996 and is part of the Norwegian public health care service [24]. The program targets about 670,000 women aged 50–69 who are invited to two view mammography, biennially. During the period from 2017 to 2021, the average attendance rate was 75%, the recall rate was 3.3%, and the rate of screen-detected cancer was 0.64%. The interval cancer rate has been stable at 0.18% since the startup of the program.

We conducted a retrospective cohort study including information about all women screened at four breast centers in BreastScreen Norway, at least once between 1 January 2007, and 31 December 2019. The women were followed for interval cancer two years after her last screening examination, until 1 December 2021. Data from four centers (Rogaland, Hordaland, Akershus, and Trøndelag) were chosen as these centers had collected automated mammographic density within the study period. Women included in the study were free from breast cancer before invitation, had at least one automated measurement of mammographic density, and completed the self-reported health indicator questionnaire used in the study [25]. Women participating in the To-Be 1 and 2 trials were excluded (*n* = 16,300) [26]. Further, 234 women diagnosed with breast cancer at the first screening examination were excluded as they had not been disease-free at risk.

Data from BreastScreen Norway was extracted from the Cancer Registry of Norway which administers the program. Questionnaire data were collected as a part of BreastScreen Norway and are described in detail elsewhere [25].

### 2.1. Study Variables

The self-reported questionnaire provided information on known risk factors for breast cancer [25], including first and second-degree family history of breast cancer [27,28], body mass index (BMI) [29], exercise and physical activity [30,31], alcohol and smoking [32,33], age at menarche [34], pregnancy [35], use of hormone therapy (HT), oral contraception, and hormone spiral [36,37,38]. First-degree family history was defined as a mother, sister, or daughter with a breast cancer diagnosis, and second-degree family history as a grandmother, aunt, or niece and no first-degree. BMI was calculated as each woman’s weight in kilograms divided by height in meters squared. Physical activity was specified as light walking, cycling, gardening, and clearing snow, while exercise was defined as regular, high-intensity activities for at least half an hour each time. Alcohol consumption was collected as the average monthly consumption of units of alcohol. Smoking was defined as having smoked at least once a week for 6 months or more. Age at menarche was specified as age at first menstruation. Pregnancy was described as the number of pregnancies lasting for more than 6 months. The use of HT, oral contraception, and hormone spiral were collected as dichotomous variables. Information on previous benign breast disease was obtained from the questionnaire and from the screening database.

Mammographic density was recorded using automated software (Volpara, version 1.5.1; Volpara Solutions, Wellington, New Zealand) [39,40]. Each examination was categorized based on Volpara density grade (VDG) 4th edition using the percent density of fibroglandular tissue: VDG1, less than or equal to 4.4%, VDG2 between 4.5% and 7.4%, VDG3 between 7.5% and 15.4%, and VDG4 equal or higher than 15.5%. VDG is designed to correlate with BI-RADS (Breast Imaging-reporting and Data System) 4th edition [41]. We included both screen-detected and interval cancers, so women were followed for at least two years after their last mammogram. Both invasive breast cancers and DCIS were included in the analysis.

### 2.2. Statistical Analysis

Age, BMI, age at menarche, mammographic density, first and second-degree family history of breast cancer, benign breast disease, exercise, physical activity, alcohol consumption, smoking, pregnancy, and use of HT, oral contraception, and hormone spiral were tested by backward selection for inclusion in the model. By using backward selection, we ensured including the minimal reasonable number of risk factors that maximized model performance. We tested interactions between the risk factors included in the model. Because none of the interaction terms were significant, they were not included in the model. The proportional hazards assumption was assessed by testing the independence between Schoenfeld residuals and time [42,43].

The means with standard deviations (SD) for quantitative variables (age, BMI, and age at menarche) and numbers with percentages for qualitative variables (mammographic density, family history of breast cancer, benign breast disease, alcohol habit, exercise, pregnancy, and use of HT) were compared for women with and without breast cancer. We used partly conditional Cox proportional hazards regression to estimate the adjusted hazard ratios (aHR) of breast cancer incidence for the aforementioned risk factors [43,44]. The Huber sandwich estimator was used to obtain robust standard 95% confidence intervals (CI) [44]. The follow-up time for each woman was defined as the time in years from the date of the first screening examination during the study period until breast cancer diagnosis or end of follow-up. Further, the same model was used to estimate aHR of breast cancer incidence for transformed from quantitative into qualitative age (50–54, 55–59, 60–64, and 65–70 years), BMI (<22; 22–25; 25–28; and >28 kg/m^2^), age at menarche (<12; 12; 13; and >13 years) and other qualitative variables (mammographic density, family history of breast cancer, benign breast disease, alcohol consumption, exercise, pregnancy, and use of HT) in order to present the estimates in a more common way for studies on breast cancer risk factors [10,12,34,45].

Following the method described by Zheng and Heagerty [42], we predicted the 4-year absolute breast cancer risk estimates using a general hazard function based on the 4-year time horizon, the length of follow-up, and each woman’s risk profile. To evaluate the performance of the model, we assessed its calibration and discrimination by internal validation. To assess calibration, we estimated the expected-to-observed (E/O) breast cancer rate ratios. The observed rate was analyzed using the Kaplan-Meier estimator to consider right censoring [46]. To assess discrimination, we estimated the area under the receiving operating characteristic curve (AUC) using the predicted 4-year risk and each woman’s outcome [47]. The AUC stands for the percentage of women with cancer who have a higher estimated risk than women without cancer. Both validation statistics and their 95% CIs were estimated using the bootstrap resampling method with 1000 samples [48].

To present the effect of the study variables in the risk estimation, we plotted the difference in the 4-year predicted risk estimated by the model with and without each variable for each woman in the population.

Statistical tests were two-sided and all *p*-values <0.05 were considered statistically significant. All analyses were performed using the statistical software R version 4.1.2 (Development Core Team, 2014).

## 3. Results

The study cohort included 73,945 women screened during the study period. After exclusions, the study cohort comprised 57,411 women who underwent 182,812 examinations during the study period. A total of 1230 breast cancers were diagnosed (Table 1). The mean follow-up time was 6.5 years.

Mean age at menarche was 13.16 years for women with breast cancer and 13.24 years for women without breast cancer (*p* = 0.037) (Table 1). No difference in mean BMI was observed for women with versus without breast cancer (25.89 versus 25.75 respectively, *p* = 0.154). Women diagnosed with breast cancer had a higher proportion of mammographically dense breasts at baseline, a higher proportion of second- and first-degree family history of breast cancer and benign breast disease, higher alcohol consumption, and a higher proportion of HT use compared with women without breast cancer. We found a higher proportion of women reporting high levels of exercise and the number of pregnancies among those without a breast cancer diagnosis.

In the adjusted model, a backward selection was performed. Smoking, physical activity, oral contraception use, and use of hormone spiral were not included in the final model due to a lack of statistical significance. All the variables in the final model were associated with the risk of breast cancer, aHR = 1.01, 95% CI: 1.00–1.03 for age, aHR = 1.06, 95% CI: 1.04–1.08 for BMI, and aHR = 0.95, 95% CI: 0.91–1.00 for age at menarche. Women with mammographic dense breasts (VDG4) had a higher risk of breast cancer (aHR= 1.71, 95% CI: 1.33–2.20) compared with those classified with VDG2. A higher risk was also observed for women with a first-degree family history of breast cancer (aHR = 1.34, 95%CI: 1.10–1.63) compared with those without, and for those with versus without a prior benign breast disease (aHR = 1.53, 95% CI: 1.31–1.78). Women who reported more than 4 h exercise per week had an aHR of 0.65 (95% CI: 0.51–0.83) using those who reported no exercise as reference. Finally, breast cancer risk was higher in women who reported the use of HT versus those who did not (aHR = 1.30, 95% CI: 1.13–1.48) (Table 2). The proportional hazards assumption was well-founded for all predictive variables (*p* = 0.76). After transformation into a qualitative variable, the effect of age at menarche was no longer significant (aHR = 1.23, 95%CI 0.99–1.54 for <11 years; aHR = 1.15 95%CI 0.96–1.37 for 12 years and aHR = 1.14, 95%CI 0.97–1.34 for 13 years, using 14 or more years as reference) (Supplemental Table S1).

**Figure 1:**
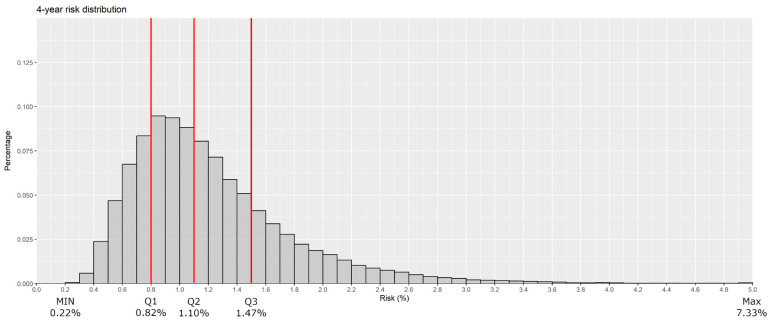
Distribution of the 4-year absolute breast cancer risk estimates. Red lines represent the quartiles of the estimated risk (MIN, minimum, Q1 = first quartile, Q2 = second quartile (median), Q3 = third quartile, MAX = maximum).

The 4-year predicted risk of breast cancer ranged between 0.22% and 7.33% (Figure 1). The three thresholds for the quartiles of four risk groups, with 25% of the population in each group, were 0.82%, 1.10%, and 1.47%. For the highest-risk group, a right-skewed distribution indicating a wide range of the risk of breast cancer was shown. The ratio between the 3rd and 1st absolute risk quartiles was 1.78 (95% CI: 1.77–1.79). We found 95% of the women to have a risk between 0.55% and 2.31%, with a ratio between the highest-risk and the lowest absolute-risk women within this group of 4.21 (95% CI: 4.17–4.26). Overall, the model slightly overestimated the risk with an E/O ratio of 1.10; (95% CI: 1.09–1.11) and the AUC was 62.6% (95% CI: 60.5–65.0%).

**Figure 2:**
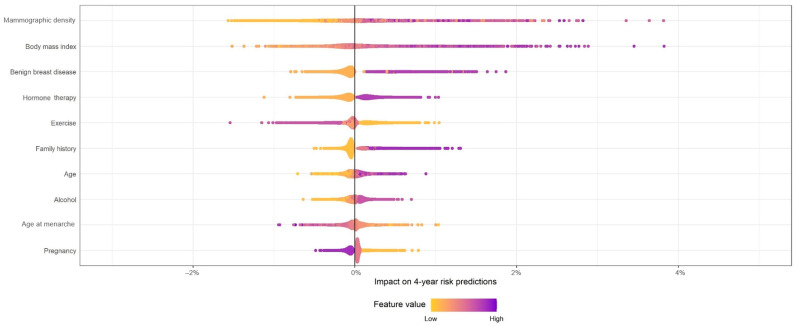
Impact on subtracting each variable from the model on 4-year absolute risk predictions. Footnote: This figure shows the impact of each variable on the different risk estimates. For each variable (*Y*-axis) and each woman (dot) in the population, the difference in the 4-year risk on the *X*-axis was estimated with the final model, and the 4-year risks estimated with the same model but without the selected variable plotted. The color of the dot represents each woman’s value in that specific variable; yellow represents low values and purple high values. For example, in the second row (body mass index) the rightmost point represents women whose risk of breast cancer is 3.88% higher when their body mass index is included in the model versus when it is not included.

We found the highest overall effect in the estimated risks of mammographic density, modifying the estimated 4-year risk of women between a 1.61% reduction and a 3.83% increase depending on her mammographic density (Figure 2). The purple colors represent higher values of each variable, indicating the risk difference when considering mammographic density increased for women with denser breasts and decreased for women with fatty breasts. This is not strictly true for each woman, because the effect of each variable in the risk prediction in absolute terms also depends on all other risk factors analyzed. The variable with the second highest overall effect in risk prediction was BMI, ranging from a decrease of 1.53% to an increase of up to 3.88% in the 4-year absolute risk prediction. Previous benign breast disease, use of HT, and a family history together with increasing age and alcohol consumption were also associated with an increase in the 4-year absolute risk prediction. On the other hand, women with higher values for exercise had a reduction in the 4-year absolute estimated risk by up to 1.56% and no activity an increase of up to 1.06%, corroborating the protective effect of this variable. Higher values of age at menarche and pregnancy also implied a reduction in the 4-year absolute estimated risk.

## 4. Discussion

We used data from a longitudinal cohort of 57,411 women to develop and validate a 4-year risk prediction model for breast cancer among women participating in BreastScreen Norway. The model included age, family history, mammographic density, BMI, previous benign breast disease, exercise, alcohol consumption, every use of hormone therapy (HT), age at menarche, and pregnancy. The wide variation in the absolute predicted risks, from 0.22% to 7.33%, contributes to substantiating the need to use such models to personalize breast cancer screening.

Similarly to other studies, we found a higher risk of breast cancer among women with mammographically dense versus non-dense breasts, also after adjusting for BMI [49], suggesting that more intensive screening strategies might be offered to women with dense breasts [50]. No difference in risk was found for mean baseline BMI between women with and without breast cancer. However, the longitudinal adjusted model showed that for each unit of difference in BMI, the risk of developing breast cancer increased by 6%, which is supported for postmenopausal women in other studies [51,52]. Our results supported studies reporting an increased risk of breast cancer for women with a prior benign breast disease [53,54] and a family history of breast cancer [27]. A protective effect of high-intensity exercise [30] and an increased risk of alcohol consumption has been shown in other studies [32]. Alcohol consumption was not statistically significantly associated with breast cancer risk in our study but was included in the model as this factor increased the discriminatory power of the model. Hormonal factors, such as age at menarche, pregnancy, and HT use have also been shown to affect breast cancer risk [34,35,36] and were included in the model due to the discriminatory power.

The E/O ratio showed that the model slightly overestimated the risk in the population, (E/O ratio = 1.1), thus, a calibration before implementation is required. The discrimination of the model was modest in our study, (AUC = 62.6%), and comparable to other studies in breast cancer risk prediction [17]. AUC could be slightly improved by adding some other risk factors that could increase the discriminatory power of the model, but it is known that a modest AUC does not detract from the usefulness of breast cancer risk prediction models [55]. However, it is argued that AUC statistics have limitations regarding the accuracy of risk prediction for low-incidence diseases [56].

When plotting the 4-year risk, we found a broad range of values in the risk estimates, reflecting the differences in risk among women targeted for breast cancer screening. These differences highlight the ability of risk prediction models to identify women at higher and lower risk compared with the average, and their need for implementing personalized screening strategies. Offering risk-based strategies to women with broad differences in the risk of breast cancer could improve the efficiency of screening [8], diminishing the proportion of false positives and detecting breast cancer earlier.

Two clinical trials are currently underway to demonstrate the increased efficiency of personalized screening, one in Europe [57] and one in the U.S. [58,59]. Both trials use robust models, which have been validated several times, however, risk models specifically designed for screening populations are needed to make better classification of risk groups and to avoid biases arising from using models developed in the clinical setting. Calibration of the model parameters should be performed prior to the implementation of risk prediction models to stratify populations. Insufficiently calibrated models may lead to biased estimates and inaccurate proportions of women classified as high or low risk. This is why, whenever possible, a model specific to the actual population is ideal, if the model is robust and validated. This is the first model specifically developed to stratify women attending the screening at BreastScreen Norway. Comparison with other well-established models would not be possible as these models would need to be calibrated to the incidence in Norway and to its population. Future studies aimed to compare calibration and the discriminatory power with those of other models would be desirable.

To our knowledge, most models on breast cancer risk prediction have not included lifestyle variables, such as exercise or alcohol habits, despite being already proven risk factors for breast cancer [30,32]. These variables are commonly difficult to obtain exhaustively but as shown in this study, they increase the discriminatory power of the models. The impact of each variable on the estimation was analyzed by means of a novel Effect plot. We plotted the risk contributed by each variable to each woman, allowing us to observe which variables had the greatest effect on the absolute risk estimates in our population. Some of the first studies on personalized breast cancer screening [5,6] suggested strategies that were not based on absolute risk, but rather on risk factors. For example, stratifying women according to their mammographic density, or after a benign breast disease. This is a more pragmatic way of designing personalized strategies. A graph representing which variables have a higher or lower effect on the risk estimation might help decide which variables are relevant for the model, and how to use the different categories for stratification.

Results from our model add knowledge about risk prediction for breast cancer among asymptomatic women participating in BreastScreen Norway and can be used to classify women into low, average, and high-risk groups which could benefit from being offered personalized screening intervals or modalities. Despite previous studies that have shown that risk-based screening strategies for breast cancer could be cost-effective, future research is needed to find the most cost-effective criteria for classifying women into different risk groups [5,6]. Previous studies suggested that low-risk women should be screened every 3 to 4 years and that high-risk women could benefit from annual screening [8]. According to our results, high mammographic density and BMI, previous benign breast disease, and family history of breast cancer were associated with the highest risk estimates. The combination of these factors in a woman would imply offering this woman a shorter screening interval and additional screening modalities, including ultrasound, contrast-enhanced mammography, and/or MRI if our personalized risk prediction model was implemented. The offer aimed to detect the cancer in an earlier stage, and thus favorable prognosis for the actual women. Alternatively, the combination of low mammographic density, normal BMI, the absence of benign breast disease, and family history of breast cancer in a woman might suggest offering this woman a 3- to 4-year screening interval using conventional screening modalities.

We used individual data from women participating in a population-based screening program for breast cancer and several risk factors, and variables related to previous screening examinations were available. This is a strength of the study. Mammographic density was automatically measured by software, reducing the risk of measurement bias. This study has several limitations. Most of the risk factors were self-reported, which could lead to information bias. However, self-reporting is a common source of information in risk prediction models [60,61]. Further, data on hormonal therapy were collected historically without information on current use, personal history of benign lesions did not include its histological type, and no information on genetic factors, which have been included in other risk prediction models, were available [62,63]. Information about menopausal status was not included in our model as we assumed that most of the women aged 50 years or older were postmenopausal in our study population. Other limitations were related to a lack of adjustment for competing risks, such as non-breast cancer death and lack of information about histopathological types of benign lesions. Although adjustment for competing risks would have been desirable, its effect on breast cancer incidence might be limited in this study as we solely included women aged 50 to 69 years. All follow-up losses were considered right censoring. Our study was performed with data from women participating in BreastScreen Norway and to verify the predictive power, the model should be tested on other study populations. Despite the fact that age at first birth is more predictive for breast cancer risk than the number of pregnancies, solely data on the latter were included in the study as the age at first birth was not available in the received dataset. Further, this study was not designed to be used to discriminate by ethnic groups, since the Norwegian population is very homogeneous and the data on ethnicity was not collected. We did not include height, as a breast cancer risk factor, in addition to BMI as our model was attributed to the population of screened women, and not to women with genetic mutations, for whom the significance of height was indicated [45,64,65].

## 5. Conclusions

The risk prediction model developed and validated showed differences in the risk of breast cancer, supporting personalized screening, which is expected to improve the risk-benefit balance of organized screening.

## Figures and Tables

**Table 1:** Characteristics of the study population.

	No Breast Cancer	Breast Cancer	*p*-Value
	*n* = 56,181	*n* = 1230	
**Age at baseline, mean [sd]**	58.15 (5.71)	56.46 (5.03)	<0.001
**Baseline BMI, mean [sd]**	25.75 (4.10)	25.89 (3.93)	0.154
**Age at menarche, mean [sd]**	13.24 (1.42)	13.16 (1.42)	0.037
**Baseline mammographic density (%)**			
VDG1	17,177 (31.02)	251 (20.41)	
VDG2	21,193 (38.54)	458 (37.24)	
VDG3	14,643 (26.79)	408 (33.17)	
VDG4	3168 (5.84)	113 (9.19)	<0.001
**Family history of breast cancer (%)**			
No	43,600 (79.17)	880 (71.54)	
Yes, 2nd degree	7252 (13.25)	192 (15.61)	
Yes, 1st degree	5329 (9.77)	158 (12.85)	<0.001
**Benign breast disease (%)**			
No	46,940 (83.55)	940 (76.42)	
Yes	9241 (16.44)	290 (23.58)	<0.001
**Alcohol consumption (%)**			
No	8536 (15.52)	182 (14.80)	
Yes, 5 or less units/month	14,429 (26.19)	284 (23.09)	
Yes, 6–10 units/month	14,212 (25.84)	305 (24.80)	
Yes, more than 10 units/month	19,004 (34.64)	459 (37.32)	0.052
**Exercise (%)**			
Never	16,259 (29.65)	396 (32.20)	
0–1 h/week	13,227 (24.04)	280 (22.76)	
2–3 h/week	19,812 (36.03)	430 (34.96)	
More than 4 h/week	6883 (12.47)	124 (10.08)	0.025
**Pregnancy (%)**			
Never	4712 (8.62)	129 (10.49)	
1 or 2	29,400 (53.48)	643 (52.28)	
3 or more	22,069 (40.10)	458 (37.24)	0.023
**Ever use of HT (%)**			
No	34,393 (62.40)	666 (54.15)	
Yes	21,788 (39.79)	564 (45.85)	<0.001

Differences in qualitative variables were tested by the chi-squared test. Differences in quantitative variables were tested by the Mann-Whitney U test. BMI = Body mass index, HT = Hormone therapy, VDG = Volpara density grade, sd = standard deviation.

**Table 2:** Partly conditional Cox proportional hazards model results with adjusted hazard ratios for the risk factors for breast cancer.

	Women-Years	aHR (95% CI)
**Age (years)**	375,078	1.01 (1.00–1.03)
**BMI (kg/cm^2^)**	375,078	1.06 (1.04–1.08)
**Age at menarche (years)**	375,078	0.95 (0.91–1.00)
**Baseline mammographic density**		
VDG1	126,951	0.59 (0.51–0.69)
VDG2	137,639	Ref.
VDG3	93,305	1.37 (1.20–1.56)
VDG4	17,182	1.71 (1.34–2.20)
**Family history of breast cancer**		
No	289,289	Ref.
Yes, 2nd degree	50,082	1.17 (0.98–1.41)
Yes, 1st degree	35,707	1.34 (1.10–1.63)
**Benign breast disease**		
No	319,891	Ref.
Yes	53,430	1.53 (1.31–1.78)
**Alcohol consumption**		
No	59,153	0.94 (0.76–1.16)
Yes, 5 or less units/month	94,432	Ref.
Yes, 6–10 units/month	93,789	1.06 (0.88–1.29)
Yes, more than 10 units/month	127,704	1.14 (0.96–1.36)
**Exercise**		
Never	104,381	Ref.
0–1 h/week	88,429	0.80 (0.67–0.96)
2–3 h/week	135,002	0.83 (0.70–0.97)
+4 h/week	47,266	0.65 (0.51–0.83)
**Pregnancy**		
Never	31,225	1.10 (0.88–1.38)
1 or 2	188,424	Ref.
3 or more	155,429	0.91 (0.79–1.04)
**Ever use of HT**		
No	226,166	Ref.
Yes	148,912	1.30 (1.14–1.49)

aHR: Adjusted Hazard Ratio, 95% CI: 95% Confidence Interval, VDG = Volpara density grade. HT = Hormone Replacement Therapy, BMI = Body mass index.

## Data Availability

Data used in the analyses can be made available on request to https://helsedata.no/ (accessed on 8 September 2023), given legal basis in Articles 6 and 9 of the GDPR and that the processing is in accordance with Article 5 of the GDPR.

## Supplementary Materials

The following supporting information can be downloaded at: https://www.mdpi.com/article/10.3390/cancers15184517/s1, Table S1: Partly conditional Cox proportional hazards model results with adjusted hazard ratios for the risk factors for breast cancer after transforming age, body mass index (BMI) and age at menarche into qualitative variables.
