# Supplementary material for: Personalized Breast Cancer Screening: A Risk Prediction Model Based on Women Attending BreastScreen Norway
Source: Cancers (Basel). 2023 Sep 12;15(18):4517. doi: 10.3390/cancers15184517 (PMC10526465; doi:10.3390/cancers15184517)
Supplement: Supplementary file 1 [file cancers-15-04517-s001.zip › cancers-2470114-supplementary.pdf]

Supplemental Table S1: Partly conditional Cox proportional hazards model results with adjusted hazard ratios for the risk factors for breast cancer after transforming age, body mass index (BMI) and age at menarche into qualitative variables

	Women-years	aHR (95% CI)
**Age (years)**		
50-54	74 742	Ref.
55-59	108 652	1.18 (1.07-1.31)
60-64	107 855	1.20 (1.03-1.38)
65-70	83 828	1.15 (0.95-1.39)
**BMI (kg/m^2)**		
<22	66 231	0.76 (0.63-0.91)
22-25	117 704	Ref.
25-28	95 645	1.41 (1.21-1.63)
>28	95 498	1.46 (1.22-1.74)
**Age at menarche (years)**		
11 or less	36 894	1.23 (0.99-1.54)
12	73 701	1.15 (0.96-1.37)
13	105 894	1.14 (0.97-1.34)
14 or more	158 589	Ref.
**Baseline mammographic density**		
VDG1	126 951	0.62 (0.53-0.71)
VDG2	137 639	Ref.
VDG3	93 305	1.39 (1.22-1.58)
VDG4	17 182	1.81 (1.41-2.32)
**Family history of breast cancer**		
No	289 289	Ref.
Yes, 2nd degree	50 082	1.17 (0.97-1.40)
Yes, 1st degree	35 707	1.34 (1.09-1.63)
**Benign breast disease**		
No	319 891	Ref.
Yes	53 430	1.53 (1.31-1.79)
**Alcohol habit**		
No	59 153	0.95 (0.76-1.17)
Yes, 5 or less units/month	94 432	Ref.
Yes, 6-10 units/month	93 789	1.05 (0.87-1.27)
Yes, more than 10 units/month	127 704	1.12 (0.94-1.34)
**Exercise**		
Never	104 381	Ref.
0-1hours/week	88 429	0.80 (0.67-0.96)
2-3hours/week	135 002	0.82 (0.70-0.96)
+4hours/week	47 266	0.65 (0.51-0.83)
**Pregnancy**		
Never	31 225	1.10 (0.88-1.38)
1 o 2	188 424	Ref.
3 or more	155 429	0.90 (0.78-1.04)
**Ever use of HT**		
No	226 166	Ref.
Yes	148 912	1.29 (1.13-1.47)
